# Posteromedial plate application using medial midline incision for complex tibia plateau fractures: a retrospective study

**DOI:** 10.1186/s12891-022-05087-1

**Published:** 2022-02-09

**Authors:** Mehmet Salih Söylemez, Serdar Kamil Cepni, Bahattin Kemah, Suat Batar

**Affiliations:** Umraniye Training and Research Hospital, Department of Orthopaedics and Traumatology, Health Sciences University, Yaprak street, Acıbadem district, No 32, D:12, 34660 Istanbul, Turkey

**Keywords:** Tibia fracture, Posterior column, Medial incision, Posteromedial plate, Internal fracture fixation

## Abstract

**Background:**

Application of a posterior plate for tibia plateau fractures associated with posterior column involvement is becoming a widespread standard practice as previous studies have shown that additional fixation of the posterior column with a posteromedial buttress plate creates strongest fixation in terms of fracture stabilization This study evaluated the clinical and radiological results of patients undergoing surgery for complex tibial plateau fractures involving the posterior column with a posteromedial plate applied via a medial midline incision.

**Methods:**

Medical records of patients undergoing surgery for Schatzker type IV, V, and VI tibia plateau fractures involving the posterior column in our institution were reviewed retrospectively. Patients with a follow-up of less than 1 year, pathological fractures, posterolateral column fractures requiring separate fixation, and open fractures were excluded from the study. Three-dimensional computed tomography (3D CT) was performed in all patients before surgery. The study population consisted of 25 patients (21 males and 4 females) with a mean age of 41.5 (19–66) years. The etiologies of the fractures were traffic accidents in seven cases, pedestrian falls in five cases, falls from a height in seven cases, and motor vehicle accidents in six cases.

**Results:**

The mean follow-up period was 15.9 months (12–25), mean time to union was 14.32 (9–20) weeks, mean Knee Society score (KSS) was 88 (81–95), and range of movement (ROM) was 123° (95°–140°). Loss of reduction was detected in only one patient (4%). A superficial incisional infection occurred in an anterolateral incision in only one patient (4%), and it recovered after oral antibiotic therapy. None of the patients required early implant removal and none had vascular or nerve complications in the postoperative period. Postoperatively, 23 (92%) patients had anatomical reduction and 2 (8%) had acceptable reduction in the sagittal plane CT sections. Acceptable reduction was achieved in 6(24%) patients and anatomical reduction was achieved in 19 (76%) in the coronal plane CT sections (Table 2).

**Conclusions:**

Clinical results of posteromedial plate application using a single medial midline incision is promising as complication rates were very low and knee scores were high.

## Background

Tibial plateau fractures are often complex injuries caused by high-energy trauma. Although the Schatzker [1] and AO Foundation/Orthopaedic Trauma Association (AO/OTA) [2] classifications are widely used for the classification and management of tibia plateau fractures, these classifications are insufficient to determine the severity of the fracture, particularly in patients with posterior column fractures after high-energy trauma. As Schatzker and AO/OTA classifications consider the fractures on the coronal plane, they direct the treatment accordingly and recommend application of plates from medial and lateral sides for fixation. However, leaving the posterior column unfixed may result in displacement of the fracture during follow-up. Therefore, tibial plateau fractures are now commonly classified according to the three-column theory using three-dimensional computed tomography (3D CT) together with the Schatzker classification, and fixation is planned accordingly [3, 4].

Posterior and/or posteromedial column fractures (AO/OTA 41 B and C/Schatzker IV, V, VI) should be considered unstable, as fractures in this area tend to undergo displacement even at low flexion angles in contrast to anterior column fractures, although they may initially appear to be non-displaced after injury [5]. Traditionally, a single anterior midline incision or anterolateral and anteromedial incisions are used for medial and lateral plating. However, these approaches do not allow adequate reduction and fixation of posterior, posterolateral, and posteromedial fragments. Therefore, several approaches that allow reduction and plate and/or screw placement in the posteromedial tibial corner have been defined. However, there is no consensus on which approach yields the best results [6]. This study evaluated the clinical and radiological results of patients undergoing surgery for complex tibial plateau fractures involving the posterior and/or posteromedial column with application of a posteromedial plate via a medial midline incision.

## Methods

All patients treated for tibia plateau fracture involving the posterior column via a medial midline incision with posterior column fixation using a plate between 2017 and 2020 in our institution were included in this study. After obtaining institutional ethics committee approval (ID: B.10.1.TKH.4.34.H.GP.0.01/372) medical records of the patients were reviewed retrospectively. This study was conducted in accordance with principles for human experimentation as defined in the Declaration of Helsinki. Informed consent was obtained from all individuals prior to surgery.

Inclusion criteria were as follows: patients undergoing operations for tibia plateau fracture involving the posterior column via a medial midline incision with posterior column fixation or buttressing using a plate, patients with the necessary medical records, including radiographic images and follow-up data, at least 12 months of follow-up, and skeletal maturity (≥ 16 years of age). Exclusion criteria were as follows: posterolateral column fracture treated through a reverse L-shaped incision in the prone position, patients with pathological fractures, open fractures, lack of sufficient medical data, < 12 months of follow-up, and patients yet to reach skeletal maturity (< 16 years of age).

3D CT was performed preoperatively to evaluate the nature of the fracture and a CT was performed postoperatively to evaluate the success of the fixation in all patients. Fractures were classified using the three-column classification system as described by Zhu et al. [7]. An axial CT section at the plateau level showing the fibular head was evaluated for classification. In this classification, three lines intersecting at the midpoint between the two eminences divided the plateau into three columns. The first line started from the tibial tuberosities, the second started from the anterior cortex of the fibular head, and the third started from the medial-posterior ridge of the tibial plateau. Medial and lateral columns were separated by the first line, and the posterior column was separated from them by the second and third lines. Luo et al. [8] modified this classification, and referred to fractures medial to the posterior tibial sulcus and those lateral to the posterior tibial sulcus as posteromedial and posterolateral column fractures, respectively. Operations were guided by this classification and fixation of each column with an individual plate was performed. Fractures involving the medial, posteromedial, or posterior column (Schatzker type IV, V, and VI) were fixed with the same technique using the same single medial midline incision. For three-column fractures, patients were treated using the routine anterolateral and medial midline approach. However, patients with a posterolateral column fracture requiring separate fixation were treated through a posterior reverse L-shaped incision in the prone position. All patients underwent surgery when the periarticular edema at the knee was alleviated and skin was ready for incision. All patients received low-molecular-weight heparin (Clexane 4000 anti-Xa; Sanofi Aventis, Paris, France) starting from the first day of admission. Antibiotic prophylaxis (1 g cefazolin for patients < 70 kg, 2 g cefazolin for patients > 70 kg) was started 30 min prior to surgery, with two additional doses administered on the day after the operation. All operations were performed by the same senior surgeon.

### Surgical technique

**Fig. 1:**
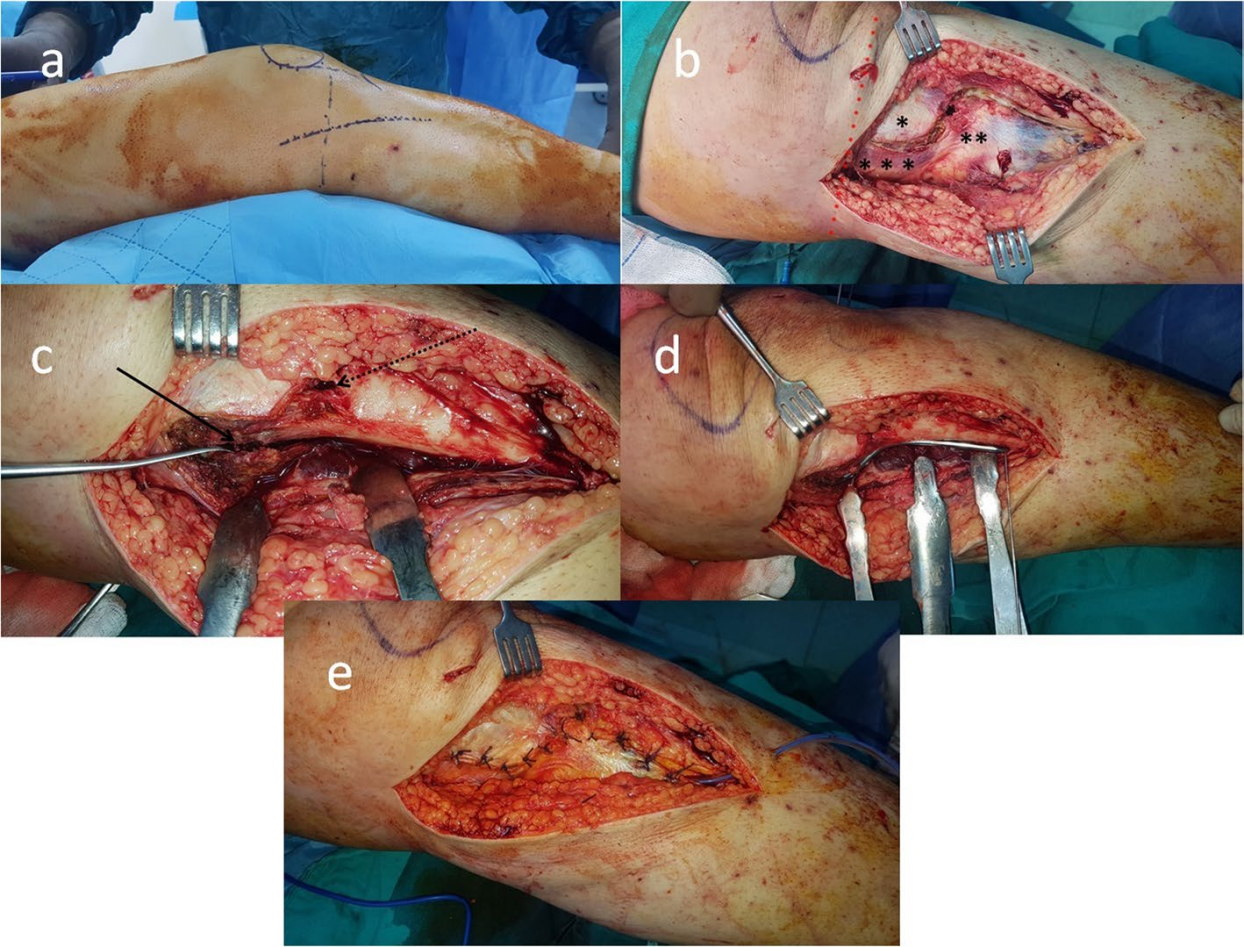
**a** Positions of anterolateral and medial midline incisions. **b** Z-shaped deep periosteal incision. *Distal attachment of the MCL to the tibia. **Pes anserinus. ***Posteromedial capsule. ****Tibia plateau. **c **Solid arrow: Distally displaced posterior column fracture. Dotted arrow: Drill hole used to elevate the lateral column fracture and shuttle the sutures for ACL avulsion fracture. **d** Plate buttressing of the posterior column. In this case, the posterior column was also fixed to the plate as medial and lateral columns were intact. **e** Posteromedial capsule and pes anserinus sutured in an anatomical fashion. The MCL tibial attachment was not dissected from its insertion. In the case of medial plating for a medial column fracture, the plate was positioned proximally on the MCL and distally under the pes anserinus

Operations were performed on a flat table, with the affected knee flexed to 30° and a padded pillow under the knee to allow clear lateral fluoroscopic imaging evaluation. A tourniquet was applied and inflated prior to starting the incision in all cases. For medial and lateral plateau fractures, separate medial midline and anterolateral incisions were performed. However, if the lateral plateau was not to be fixed, a single medial midline incision was used (Fig. 1a). An oblique medial midline incision was made, starting about 3 cm above the knee joint and extending 10 cm distal in the sagittal midline plane. After subcutaneous dissection, the pes anserinus was elevated from its insertion subperiosteally using a Z-shaped sharp periosteal incision (Fig. 1b). This subperiosteal deep incision enabled dissection of the posteromedial capsule from the plateau without disturbing the tibial insertion of the medial collateral ligament(MCL) (Fig. 1b–d). Moreover, using this incision, anatomical closure of the deep fascia and pes anserinus could be achieved (Fig. 1e). Subperiosteal dissection of the posteromedial capsule from the posterior tibial plateau enables a relatively safe zone for plate placement. After dissection of the posterior column fracture, a plate of suitable size was placed on the posterior and/or posteromedial plateau. Taking the knee in flexion and external rotation eased mobilization of the posterior skin flap and provided a sufficient field of view for both fixation and reduction. The choice of plate used for the surgery was made at the discretion of the operating surgeon. Either a 2.2 mm semitubular or a 3.5 mm dynamic compression plate (DCP) (TST Medical Devices, Istanbul, Turkey) was contoured to the shape of the posterior column and used for buttressing (Fig. 1d).

After placement of the plate, the knee was taken into extension to facilitate reduction of the posterior column. The first screw (3.5 mm cortical non-locking) was placed on the distal side of the fracture line and used to buttress the posterior plateau anteriorly. Two or three additional 3.5 mm cortical screws were placed distally to enhance stability. In 20 cases, plates were used for their buttressing effect and the posterior fragment was not fixed to the posterior plate. In five cases (three patients with isolated posteromedial column fracture, one with medial and posteromedial column fracture, and one with three-column fracture), the posterior fragment was also fixed to the posteromedial plate. The order of placement of the plates started with posterior column buttressing and proceeded with lateral column fixation and medial column fixation.

### Postoperative care and evaluation

A hinged brace allowing 0°–90° flexion was applied postoperatively. Patients were mobilized allowing toe-touch walking without weight bearing. Quadriceps strengthening and hamstring flexing exercises were started on postoperative day 1. Clinical and radiographic assessments of the progress of healing and complications were carried out during follow-up visits. Two authors assessed the preoperative and postoperative images independently. The quality of reduction was evaluated in both coronal and sagittal CT planes. Displacement of 0 mm was defined as anatomical reduction, ≤ 2 mm was considered acceptable reduction, and > 2 mm was deemed poor reduction. All patients came for routine follow-up visits at 2 weeks, 6 weeks, 3 months, 6 months, and 1 year postoperatively. Weight bearing was allowed after 3 months of follow-up if callus was evident on at least three cortices. Flexion of 90°–120° was allowed after 6 weeks of follow-up. Duration of union and loss of reduction, if any, were recorded. Range of motion (ROM) and Knee Society score (KSS) were evaluated and recorded at the last follow-up after 1 year [9]. In addition, possible complications, including deep vein thrombosis (DVT), infection, nonunion, and delayed union, were recorded.

Data were analyzed using SPSS software (ver. 22.0; IBM Corp., Armonk, NY, USA). Interobserver reliability for qualitative data was assessed using the kappa coefficient (κ). The intraclass correlation coefficient (ICC) was used to assess reliability of quantitative data between two observers. Quantitative variables are expressed as the mean or median and standard deviation, and qualitative variables are expressed as the frequency or ratio.

## Results

Operations for tibia plateau fractures involving the posterior column were performed in 32 patients. Three patients who were treated for posterolateral column fractures and four patients who were lost to follow-up were excluded from the study. Therefore, 25 patients with a mean age of 41.5 (19–66) years (21 males and 4 females) were ultimately included in the analysis. Patient demographic characteristics are summarized in Table 1.

**Table 1:** Preoperative patient demographics

Mean/±SD/Range		
**Age (years)**		41,5±12,5(19-66)
**(n/%)**		
**Side**	Right	13(52%)
	Left	12(48%)
**Trauma Pattern**	Traffic accident	7(28%)
	Pedestrian fall	5(20%)
	Motor vehicle accident	6(20%)
	Fall from a Height	7(20%)
**ASA Scores**	ASA 1	13(52)
	ASA 2	10(40)
	ASA 3	2(8)
**Schatzker Fracture pattern**	Type 4	10(40%)
	Type 5	6(24%)
	Type 6	9(36%)
**AO/OTA Fracture pattern**	41 c1	7(28/%)
	41 c2	3(12/%)
	41 c3	5(20/%)
	41b2.3	1(4/%)
	41b3.3	9(36/%)
**Three column fracture pattern**	1 column fracture	3(12/%)
	2 column fracture	7(28/%)
	3 column fracture	15(40/%)
**Accompanying fracture**	Calcaneus	2(8%)
	Distal radius	1(4%)
	lomber vertebra fracture	1(4%)
	Clavicula + Calcaneus	1(4%)

*SD *Standart Deviation*, ASA *American Association for Anesthesiologists

**Fig. 2:**
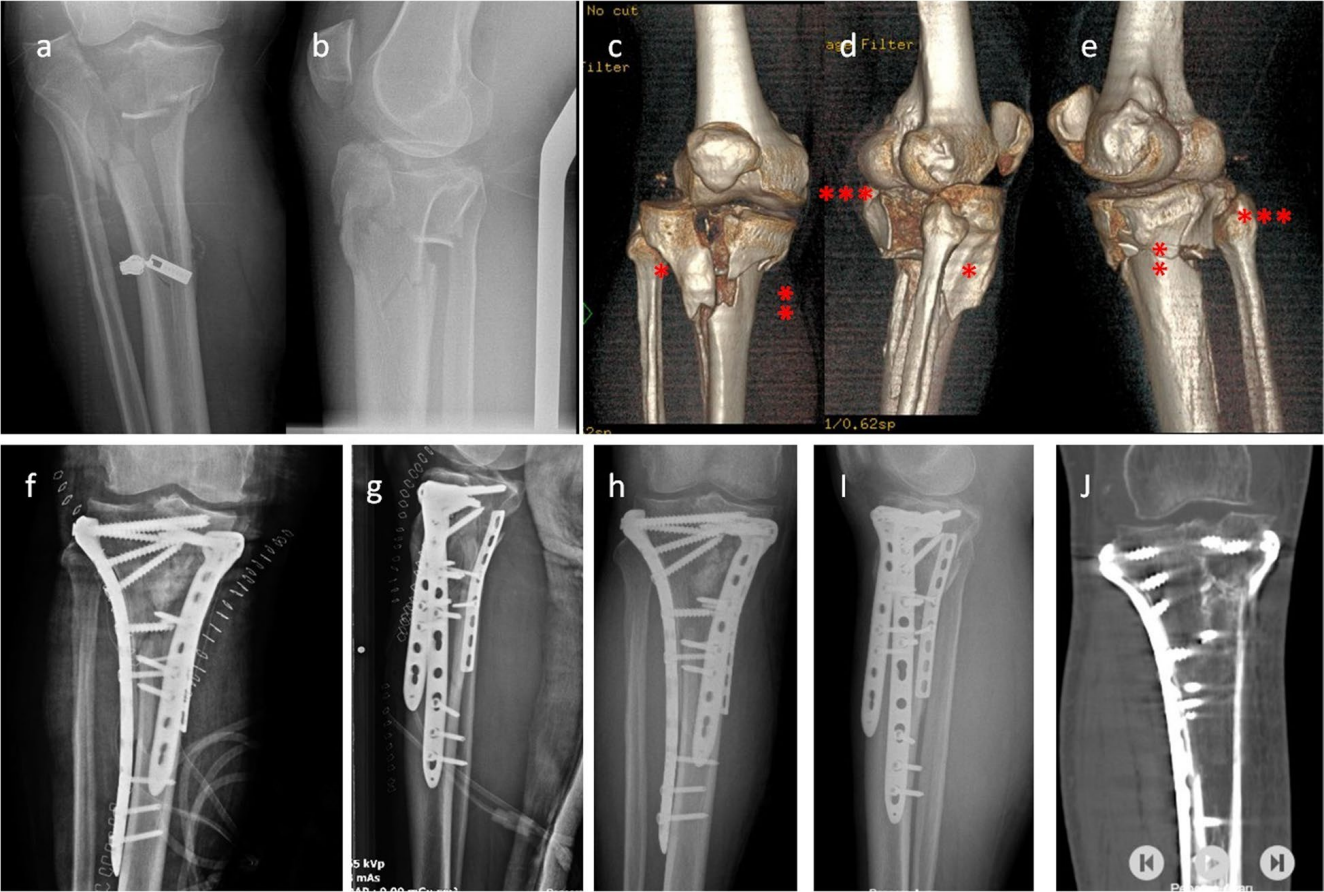
**a**, **b** A patient sustaining right tibia plateau fracture after a motor vehicle accident. **c–e** 3D CT revealed fractures of three columns. *Lateral column, **medial column, ***posterior column. **f**, **g** The patient was treated using two incisions for fixation of three columns with three plates. Acceptable reduction was obtained during surgery. **h–j** X-ray and CT at 13 months postoperatively showed perfect union of the fracture

**Fig. 3:**
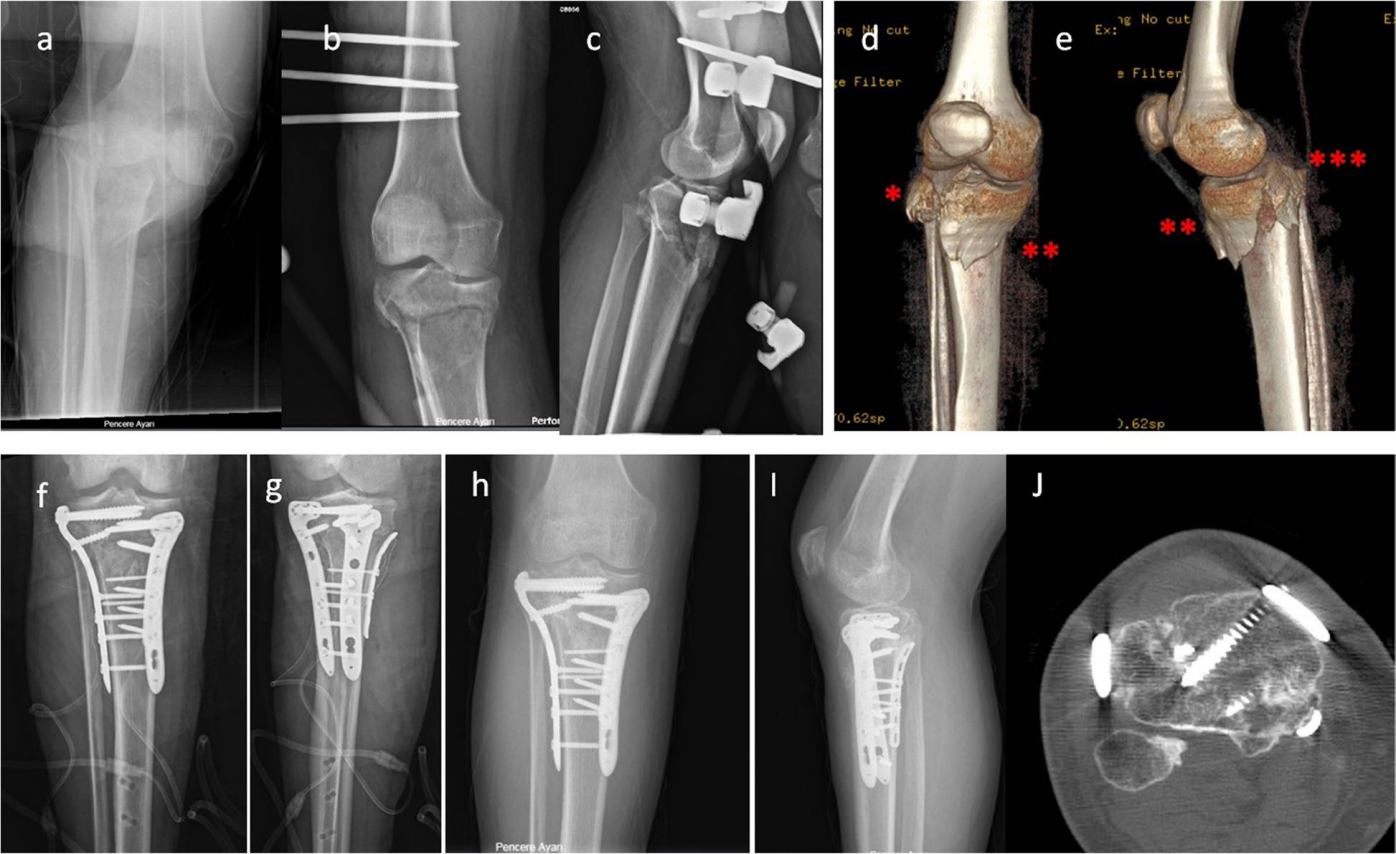
**a–c** A patient sustaining right plateau fracture after a traffic accident. Provisional reduction was obtained with provisional unilateral external fixators. **d**, **e** 3D CT revealed fractures of three columns. *Lateral column, **medial column, ***posterior column. **f**, **g** The patient underwent definitive surgery on day 21 after trauma. The patient was treated using two incisions for fixation of three columns with three plates. Anatomical reduction was obtained during surgery. **h–j** X-ray and CT at 16 months postoperatively showed perfect union of the fracture. **j** Axial CT showing fixation and union of three individual columns

Excellent agreement was observed between the two independent surgeons for evaluation of reduction quality (amount of articular step-off) and fracture classification (ICC ≥ 0.93 and κ ≥ 0.95, respectively). Fifteen patients were treated for a three-column fracture using three plates through the standard anterolateral approach with a medial midline incision (Figs. 2 and 3). In seven patients with two-column fractures, including medial and posteromedial fractures, two plates were used for fixation through a single medial midline incision. Three patients had a posteromedial column fracture associated with anterior cruciate ligament (ACL) avulsion fracture and focal depression of the posterolateral plateau. In these patients, the posteromedial column was fixed with one plate via a medial midline incision, and the lateral plateau was elevated, grafted with autologous iliac bone, and supported with percutaneously placed screws to prevent depression. ACL avulsion fractures were addressed arthroscopically using a suture shuttling technique. Four patients had a medial and three had a lateral meniscus injury, which were treated either with arthroscopic intervention or via arthrotomy incisions depending on the location and type of injury.

The mean length of surgery was 124.4 (60–165) min. Autologous iliac bone grafts were applied in 16 (64%) patients. None of the patients required transfusion postoperatively. Using this technique, postoperative CT showed that 23 (92%) patients had anatomical reduction and 2 (8%) had acceptable reduction in the sagittal plane. Acceptable reduction was achieved in 6(24%) patients and anatomical reduction was achieved in 19 (76%) in the coronal plane (Table 2).

**Table 2:** Postoperative patient characteristics

	Mean(±SD)	Minimum- Maximum	Median	Range
**Time to surgery(days)**	8,36±,6,67	2-23	6	21
**Preoperative Hgb levels**	13,016±1,38	10,4-15,7	13	5,3
**Postoperative Hgb levels**	11,252±1,32	8,9-14	11,6	5,1
**Length of surgery(minutes)**	124,4±37,8	60-175	140	115
**Postoperative Hospitalisation time(days)**	3,96±2,63	2-12	3	10
**Time to Union (weeks)**	14,32±3,41	9-20	14	11
**KSS**	88±3,94	81-95	90	14
**Knee ROM range(°)**	123±12.5	95-140	130	45

*KSS* Knee society score, *SD* Standart deviation, *Hgb* Haemoglobin, *ROM* Range of motion

The mean follow-up period was 15.9 (12–25) months, mean time to union was 14.32 (9–20) weeks, mean KSS score at 1 year postoperatively was 88 (81–95), and mean ROM at 1 year postoperatively was 123° (95°–140°). Knee extension was full and within the normal range in 22 patients, and 3 (12%) showed 5°–10° flexion contracture. There were no cases of nonunion, malunion, or delayed union. Loss of reduction was detected in a 66-year-old patient with 20° genu valgum with a medial and posteromedial (Schatzker type 4) column fracture (4%). This patient sustained 2 mm depression of the medial column after weight bearing. However, the patient achieved union without further deterioration. A superficial infection was detected in only one (4%) patient in the anterolateral incision, and it recovered after oral antibiotic therapy. None of the patients required early implant removal and none had vascular or nerve complications during the follow-up period (Table 2).

## Discussion

Posterior column fractures were first defined by Postel et al. [10] in 1974, and application of a posterior plate for tibia plateau fractures associated with posterior column involvement is becoming a widespread standard practice as previous studies have shown that additional fixation of the posterior column with a posteromedial buttress plate creates strongest fixation in terms of fracture stabilization [11–13]. In this study, we evaluated the radiological and clinical results of patients treated via a medial midline incision for plateau fractures particularly involving the posteromedial and posterior column. The technique was easy and allowed adequate visualization of the fracture and application of a posteromedial plate. It was not demanding and all patients achieved union without the need for additional surgical interventions.

Supine and prone approaches may have relative advantages and disadvantages. With the Lobenhoffer technique, it is impossible to manipulate all fragments in the case of a three-column fracture. In addition, with use of a medial approach, posterolateral column fractures may not be addressed appropriately [14–16]. Operating with the patient in the supine position allows the surgeon to intervene in all three column fractures simultaneously. For three-column fractures, the posterior column was buttressed with a posteromedial plate as the first step, and then the lateral column was elevated and/or reduced and fixed with relatively short proximal screws. As the final step, the medial column was reduced and fixed with a medial plate and screws inserted proximally from the lateral side were changed for longer screws (Fig. 3). The screws inserted from the lateral side are oriented toward the medial column and fix the medial column to the lateral column better than screws inserted from the medial plate, as these screws are oriented toward the posterolateral column rather than the lateral column. This was the reason for 2 mm displacement in a single case in our series. In this case, we had used an additional free screw inserted from the medial side going to the lateral column to compress the plateau. However, this single screw was not strong enough to prevent displacement and an additional 20° of genu valgum increased the vertical load on the medial column causing an acceptable amount of distal displacement of the medial column. In addition, after placing three plates we were able to fix the posterior column to the plate to increase the stability of the posterior column. This is impossible with posterior approaches as dynamic fixation of the posterior column with buttressing is essential until completion of reduction and fixation of the associated medial and lateral columns. With use of posterior approaches, it is impossible to intervene in the posterior column after changing the position of the patient, and if fixed to the plate in an inappropriate position, this will cause further deterioration of the medial and lateral columns. Therefore, rather than fixation of the posterior column, buttressing with a plate is the most common fixation technique for these injuries. In our series, we fixed the posterior column to the plate in four cases, and radiological and clinical results of these patient did not differ from the others despite the instability of the posterior column in these cases. Posterior approaches must be used for patients with posterolateral column fracture because accessing such fractures via a medial midline or posteromedial incision is very difficult. Although the direct posterior approach enables easy restoration of collapsed articular cartilage, it also has some disadvantages, such as superficial wound necrosis and flexion contracture [17, 18]. Some proponents of this technique have used this approach just to place a buttress plate rather than for elevation of a depressed lateral plateau or for fracture reduction, with elevation and final reduction of the lateral and posterolateral plateau using the anterolateral incision [6]. Thus, although it is inevitable that the posterior approach is used in the prone position in patients with posterolateral column fractures, using a medial midline incision in patients with posterior column or posteromedial column fractures facilitates both reduction of the posterior fragment and intervention of the injury globally.

Far-posteromedial incision is another approach that can be used for intervention in the posteromedial column with the patient in the supine position [19]. With this technique, the medial head of the gastrocnemius (MHG) is retracted laterally and the pes anserinus is retracted anteromedially. This approach does not provide a perfect view of the articular surface and accessing the medial column and application of a medial plate can be challenging. It may be speculated that using a medial midline incision and anterolateral incision together may jeopardize the circulation of the skin between incisions. However, in our series, we did not encounter any incision problems except in one patient with a superficial anterolateral incisional infection. However, there was no necrosis in this patient and the superficial infection was resolved with oral antibiotics. Zhang et al. [20] used combined lateral peripatellar and posteromedial approaches for treatment of Schatzker type IV (medial and posteromedial column) tibial plateau fractures and reported satisfying outcomes. Our series included 10 patients with Schatzker type IV fractures. Seven of these patients had medial and posteromedial column fractures. All of these fractures were treated using the same medial midline incision with two plates. For this particular fracture type, a single incision was sufficient to achieve anatomical reduction with satisfactory stable fixation. It was also very easy to perform an arthrotomy just posterior or anterior to the MCL through the same incision, and additional interventions for meniscal injuries could be performed (Fig. 1). Open reduction and internal fixation of plateau fractures through a compromised soft tissue envelope has been reported to be associated with major wound complications [21]. Therefore, sparing the soft tissue as much as possible is of the utmost importance while achieving anatomical reduction of the articular surface, as articular incongruity of the tibial plateau at a minimal level compromises long-term functional outcomes [22]. In our series, anatomical and acceptable reduction was obtained in all cases through two incisions with good functional outcomes. Loss of extension between 5° and 10° was encountered in three cases treated for three-column fractures.

There is no consensus in the literature for the ideal implant selection for fixation of posterior column fractures. While some authors had used manufactured anatomical 3.5mm T buress plates for the posterior column fixation, some authors have used semitubular plates or both together [7, 15]. Since the posteromedial cortical surface is a location with too many curves, there is no manufactured T-type buttress plate that can be applied here. For this reason, we preferred to use and contour the semitubular or DCP plates for fixation. It can be speculated that bending the plates may reduce the strength of the plates. However, in none of the cases a failure of the plate was detected at posteromedial area.

The medial midline approach also has its limitations. With a small medial midline incision, this approach may not provide the surgeon with a comprehensive direct view of the more posterior and lateral aspects of the tibial plateau that may require intervention. This approach, as in other posterior direct approaches, does not work well close to major neurovascular structures, and caution is required when retracting the MHG. The major limitations of our study were that it was not a prospective randomized study, did not have a control group, and the overall number of patients was too small to support more definitive conclusions. To confirm the effectiveness of posteromedial plating using a medial midline incision, a randomized controlled study with a larger sample size is required.

## Conclusions

Treatment of tibial plateau posterior column fractures remains challenging even for experienced surgeons. Although the involvement of the posterolateral column still requires access in the prone position, our results indicate that posteromedial plate application can be performed safely and appropriately in patients with posteromedial column fracture without making a separate posterior incision. Moreover, in these complex fractures, posterior column fixation prevents collapse of the articular surface during follow-up and provides good clinical and radiological results.

## Data Availability

The datasets used and/or analysed during the current study are available from the corresponding author on reasonable request.

## Abbreviations

3D CT: Three-dimensional computed tomography
AO/OTA: AO Foundation/Orthopaedic Trauma Association
DCP: dynamic compression plate
MCL: Medial collateral ligament
ACL: anterior cruciate ligament
DVT: deep vein thrombosis
ICC: Intraclass correlation coefficient
KSS: Knee Societ Scores
ROM: Range of Movement
